# Examining the Association Between the Extent of Anterior Longitudinal Ligament Ossification Progression and Comorbidities in Diffuse Idiopathic Skeletal Hyperostosis

**DOI:** 10.7759/cureus.51357

**Published:** 2023-12-30

**Authors:** Daiki Ishimura, Tadao Morino, Yusuke Murakami, Shintaro Yamaoka, Tomofumi Kinoshita, Masaki Takao

**Affiliations:** 1 Bone and Joint Surgery, Ehime University, School of Medicine, Ehime, JPN; 2 Orthopedic Surgery, HITO Hospital, Ehime, JPN

**Keywords:** diabetes mellitus, obesity, comorbidity, ligament ossification, diffuse idiopathic skeletal hyperostosis

## Abstract

Background: It has been documented that diffuse idiopathic skeletal hyperostosis (DISH) exhibits a higher prevalence among elderly, male, and obese individuals. Additionally, diabetes mellitus and other comorbidities are more frequently observed in this patient population. However, there is a lack of reports exploring the correlation between the extent of ossification and these demographic and clinical characteristics.

Objective: To examine the correlation between comorbidities and the severity of ossification of the anterior longitudinal ligament in patients with DISH.

Materials and Methods: The study included 468 patients who visited our hospital in 2018-2022. They were divided into DISH and non-DISH groups based on computed tomography image evaluation according to the Resnick criteria. The patients in the two groups were matched for age and sex. We compared comorbidity-related factors including body mass index (BMI), serum markers for metabolic syndrome and renal function, and hemoglobin A1c (HbA1c) levels between the matched groups. Moreover, we examined the correlation between the number of fused vertebral bodies and comorbidity-related factors, age, and sex within the DISH group.

Results: The DISH group included significantly more men and elderly patients than the non-DISH group. Furthermore, the average BMI and HbA1c levels were significantly higher in the matched-DISH group than in the matched-non-DISH group, whereas no differences were observed in other markers. In the DISH group, the number of fused vertebral bodies did not correlate with age, sex, BMI, or HbA1c levels.

Discussion: BMI and HbA1c levels were significantly higher in patients with DISH than those without; however, the number of fused vertebral bodies and the possibility of having coexisting obesity or diabetes mellitus showed no correlation with the age or sex of the patient. Therefore, each patient should be carefully assessed for ossification severity regardless of age, sex, and the comorbidities they possess.

## Introduction

Diffuse idiopathic skeletal hyperostosis (DISH) is a pathological condition characterized by progressive ossification of ligaments throughout the body and at ligament attachment sites. Within the spine, ossification of the anterior longitudinal ligament causes spinal ankylosis. This condition was reported by Forestier et al. in 1950 and subsequently termed as “DISH” by Resnick et al [1,2]. The most commonly used diagnostic criteria for the condition were developed by Resnick and Niwayama et al [3]. They proposed that DISH diagnosis should be given when the following criteria are met: (1) there is bony bridging of the anterior or lateral aspect of at least four vertebral bodies; (2) the height of the intervertebral discs is relatively preserved; and (3) there is no ankylosis of the sacroiliac joint.

The progression of anterior longitudinal ligament ossification varies in extent among patients with DISH. Some patients display bridging of only four adjacent vertebral bodies (the minimum number required to meet the diagnostic criteria), whereas others exhibit bridging of almost all 24 vertebral bodies from the cervical to sacral spine [4]. Additionally, the bridging of vertebral bodies progresses more rapidly in younger patients [5], and the number of fused vertebral bodies increases over the duration of the condition [6].

As the bridging between vertebral bodies progresses cranially and caudally, the spinal flexibility decreases. Stress shielding also decreases bone strength, therefore, the application of any minor external force is more likely to cause fractures [7-9]. In addition, when a penetrating fracture occurs in the ankylosed spine, bone union is difficult to achieve using conservative therapy, often causing delayed neurologic deficits. Thus, spinal fixation is recommended [10].

The causes of DISH are currently unknown. However, the pathological condition has been associated with numerous other conditions including lifestyle diseases (e.g., obesity, diabetes mellitus [DM], and hyperlipidemia), hypertension, and heart and brain diseases, with an increased prevalence in the elderly population and men [11-13].^ ^Additionally, the involvement of the collagen type6 alpha1 gene and other single nucleotide polymorphisms have been reported based on genetic analyses [14,15]. If instigators such as genes that encode the proteins stimulating ossification processes are involved in the development of comorbidities, patients with more advanced ossification may be more likely to have comorbidities, increasing the incidence of postoperative complications. Furthermore, it may be necessary to encourage even asymptomatic patients with DISH to undergo examination for each comorbidity if they are found to have progressive ossification.

In the current study, we examined groups without the effects of age and sex (matched groups) to determine whether DISH is associated with comorbidities that are reported to be common in patients with DISH. We also examined whether the extent of ossification progression, that is, the number of fused vertebral bodies, is associated with comorbidities and coexisting factors.

If we are aware that the probability of comorbidities increases with the advancement of ossification, it becomes imperative to give heightened consideration to comorbidities in patients displaying a pronounced tendency for ossification.

## Materials and methods

Study design

This is a retrospective observational study.

Patients

Among patients who visited our hospital and underwent computed tomography (CT) of the area between the cervical spine and the pelvis, regardless of primary diseases, between 2018 and 2022. The current study involved 468 patients who underwent hematologic tests covering necessary items within three months before and after CT. Because this study was retrospective, the patient’s informed consent was obtained by opting out. The selection of these patients was conducted utilizing a database comprising images and blood tests linked to electronic health records, from which individuals meeting the aforementioned criteria were chosen. No exclusion criteria were stipulated. We announced on the website of the Clinical Research Support Center, the Department of Orthopedic Surgery of our university hospital, and the hospital bulletin board that we would be conducting this study, and provided an opportunity to reject the use of the data. This study was approved by the institutional review board at our University Hospital (IRB approval no. 2207011).

Diagnosis of DISH

The sagittal and coronal CT images of all patients were examined based on the diagnostic criteria developed by Resnick and Niwayama et al [3]., and DISH was diagnosed when the following three conditions were met: (1) the presence of bony bridging of the anterior or lateral aspect of four or more adjacent vertebral bodies, (2) relatively preserved intervertebral disc space, and (3) the absence of ankylosis in the sacroiliac joint.

Group assignment

First, 468 patients were divided into the DISH group that met the diagnostic criteria for DISH and the non-DISH group that did not meet them. Then, patients matched for sex and age (within +/−1 year) were extracted from each group. The groups of patients extracted from the DISH and non-DISH groups were referred to as the matched-DISH and matched-non-DISH groups, respectively.

Investigation 1: comparison between the matched groups

**Figure 1:**
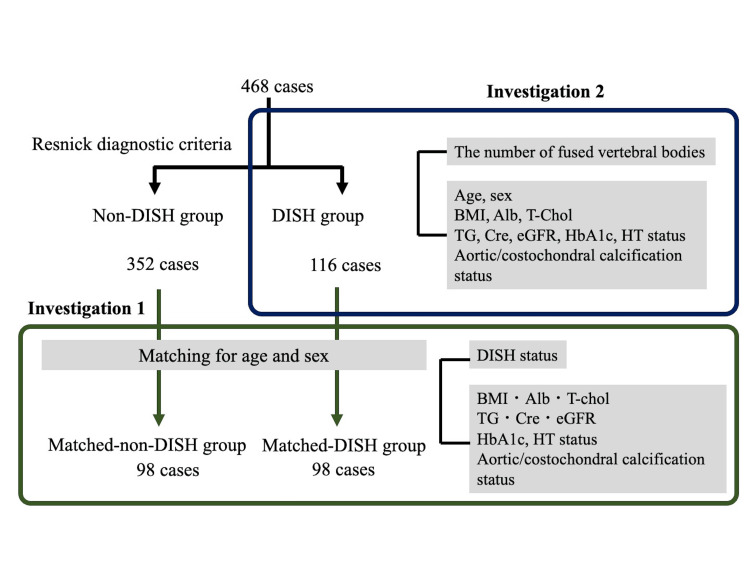
Schematic of the group assignment and the study objectives DISH: Diffuse idiopathic skeletal hyperostosis; BMI: body mass index; Alb: serum albumin; T-Chol: total cholesterol; TG: triglyceride; Cre: creatinine; eGFR: estimated glomerular filtration rate; HbA1c: hemoglobin A1c; HT: hypertension

The matched-DISH and matched-non-DISH groups were compared for body mass index (BMI) and hematologic test values (i.e., serum albumin, total cholesterol [T-Chol], triglyceride [TG], creatinine [Cre], estimated glomerular filtration rate [eGFR], and hemoglobin A1c [HbA1c] levels), and presence or absence of hypertension, aortic calcification, and costochondral calcification (Figure 1). The presence or absence of hypertension was determined by assessing the patient's history regarding hypertension and the use of oral antihypertensive agents. Aortic calcification was deemed to be present if CT images showed at least one calcified lesion in the area from the aortic arch to the descending aorta. Costochondral calcification was deemed to be present if CT images showed at least one calcified costal cartilage.

Investigation 2: correlation with the number of fused vertebral bodies

Next, in each patient within the DISH group, the number of fused vertebral bodies between cervical vertebra C2 and sacral vertebra S1 (24 vertebral bodies) (including all noncontiguous fused segments) on CT images was counted to determine the total number of fused vertebral bodies (Figure 1). We examined the correlation between the number of fused vertebral bodies and age, sex, BMI, and serum albumin, T-Chol, TG, Cre, and eGFR levels. We also examined whether the number of fused vertebral bodies differed between patients with and without hypertension, aortic calcification, and costochondral calcification. 

Statistical methods 

Statistical analyses were performed using JMP version 17.0 for Mac (SAS Institute Inc., Cary, NC). Chi-square and Student’s t-tests were performed to compare continuous and categorical variables, respectively. Spearman’s rank sum correlation coefficients were calculated to assess the presence or absence of correlation. The Mann-Whitney U test was performed to test ordinal variables. A p-value of <0.05 was considered to indicate a significant difference, and a p-value of <0.1 was considered to indicate a trend.

## Results

Background data

The mean age of all 468 patients was 60.4 ± 16.8 years, with 113 men and 355 women. Furthermore, the DISH group included 116 patients (24.8%), and the non-DISH group included 352 patients (75.2%). The mean age was 69.5 ± 10.0 years in the DISH group and 57.4 ± 17.5 years in the non-DISH group. Notably, the DISH group was significantly older (P < 0.0001) and the male-to-female ratios were 42.2:57.8 in the DISH group and 18.2:81.8 in the non-DISH group. Therefore, the proportion of men was significantly higher in the DISH group (P = 0.0001). After matching for age and sex, both matched-DISH and matched-non-DISH groups included 98 patients each.

Comparison of each factor between the matched groups

The mean age was 68.0 ± 10.0 years in the matched-DISH group and 67.8 ± 10.0 years in the matched-non-DISH group and each group included 67 men and 31 women. The mean number of fused vertebral bodies was 6.7 ± 2.7 in the matched-DISH group and 0.2 ± 0.4 in the matched-non-DISH group (P < 0.0001). Furthermore, the mean BMI was 24.8 ± 6.7 in the matched-DISH group and 21.7 ± 4.1 in the matched-non-DISH group; therefore, the matched-DISH group had a significantly higher BMI (P = 0.0003). The mean HbA1c levels were 6.7 ± 1.4% in the matched-DISH group and 6.2 ± 0.8% in the matched-non-DISH group. They were significantly higher in the matched-DISH group (P = 0.002). Although no significant differences were observed in albumin, T-Chol, TG, Cre, or eGFR between the two groups, the eGFRs were 66.9 ± 23.2 mL/min/1.73 m^2^ in the matched-DISH group and 76.3 ± 45.0 mL/min/1.73 m^2^ in the matched-non-DISH group, showing that the renal function tended to be more impaired in the matched-DISH group (P = 0.09). Moreover, no differences were observed in the presence or absence of hypertension, aortic calcification, or costochondral calcification between the two groups (Table 1).

**Table 1:** Comparison of each factor between the matched-DISH and matched-non-DISH groups DISH: Diffuse idiopathic skeletal hyperostosis; BMI: body mass index; Alb: serum albumin; T-Chol: total cholesterol; TG: triglyceride; Cre: creatinine; eGFR: estimated glomerular filtration rate; HbA1c: hemoglobin A1c * p<0.01, # p<0.1

	Matched-DISH	Matched-non-DISH	P-value
Sample size (n)	98	98	-
Sex (male:female) (n)	67:31	67:31	-
Number of fused vertebral bodies	6.7 ± 2.7	0.2 ± 0.4	-
-	-	-	(t-test)
BMI	24.8 ± 6.7	21.7 ± 4.1	0.0003^*^
Alb (g/dL)	3.6 ± 0.7	3.6 ± 0.7	0.84
T-Chol (mg/dL)	190.7 ± 41.2	203.3 ± 68.9	0.14
TG (mg/dL)	150.2 ± 74.5	144.8 ± 115.3	0.7
Cre (mg/dL)	0.9 ± 0.9	0.8 ± 0.7	0.6
eGFR (mL/min/1.73 m^2^)	66.9 ± 23.2	76.3 ± 45.0	0.09^#^
HbA1c (%)	6.7 ± 1.4	6.2 ± 0.8	0.002^*^
-	-	-	(chi-square test)
Hypertension (n)	62	54	0.25
Aortic calcification (n)	92	93	0.999
Costochondral calcification (n)	98	98	1

Association between the number of fused vertebral bodies and each factor

The mean number of fused vertebral bodies was 6.8 ± 2.8 (range 4-15) in the 116 patients included in the DISH group. However, the number of fused vertebral bodies did not significantly correlate with any of the following factors: age, sex, BMI, hematologic test values, hypertension, aortic calcification, and costochondral calcification (Table 2).

**Table 2:** Correlation between the number of fused vertebral bodies and each factor in the DISH group DISH: Diffuse idiopathic skeletal hyperostosis; BMI: body mass index; Alb: serum albumin; T-Chol: total cholesterol; TG: triglyceride; Cre: creatinine; eGFR: estimated glomerular filtration rate; HbA1c: hemoglobin A1c

-	(Mean ± SD)	Correlation coefficient (Spearman’s)	P-value
Age (y.o.)	69.5 ± 10.0	0.067	0.473
BMI	24.3 ± 6.3	0.091	0.378
Alb (g/dL)	3.6 ± 0.7	0.001	0.992
T-Chol (mg/dL)	186.9 ± 41.5	0.036	0.744
TG (mg/dL)	147.5 ± 76.3	0.049	0.642
Cre (mg/dL)	0.92 ± 0.83	−0.02	0.842
eGFR (mL/min/1.73m^2^)	66.7 ± 22.9	0.081	0.441
HbA1c (%)	6.68 ± 1.42	0.007	0.945
-	-	(Mann–Whitney U test)	
Sex (male:female) (n)	49:67	-	0.14
Hypertension (n)	66	-	0.41
Aortic calcification (n)	110	-	0.99
Costochondral calcification (n)	116	-	1

## Discussion

In the current study comparing the matched-DISH and matched-non-DISH groups of patients matched for age and sex, the matched-DISH group showed significantly higher BMI and HbA1c levels. In the DISH group, the number of fused vertebral bodies showed no correlation with age, sex, BMI, serum albumin, T-Chol, TG, Cre, eGFR, HbA1c, hypertension, aortic calcification, or costochondral calcification levels.

The correlation of advanced age with male sex has been reported in many articles [4,5,12,16-20]. In the current study, the DISH group included significantly older patients and more men than the non-DISH group, as suggested. Although the causes of DISH are unknown, it is considered that chronic inflammation may be involved in the stage before the condition onset [21] and also that mechanical stress is involved as the apical vertebra of thoracic kyphosis is frequently ossified [4]. If ossification occurs along with the upsurge of inflammation and stress with age, a higher prevalence in elderly individuals would be expected. However, the results of the current study showed no correlation between the extent of ossification progression and age. Consequentially, this finding should be taken into consideration so that presumptions are not made and care is given fairly to those with more severe conditions and is not simply based on age. Additionally, as previous reports have found, ossification progresses more rapidly in younger patients and genetic factors may be involved [5,14,15,22], it seems that severe ossification is likely to depend on an individual’s susceptibility to ossification. The same theory applies to the sex of the patient; although the prevalence of DISH was higher in men, the severity of the ossification was not. Therefore, this suggests that the individual susceptibility to severe ossification is also not associated with the sex of the patient.

Regarding the association of DISH with the BMI of the patient, many articles have reported that the average BMI was higher in patients with DISH than those without. For instance, Harlianto et al. [19] compared DISH and non-DISH groups adjusted for age and sex in a cohort study involving 4624 patients and reported that the average BMI was significantly higher in the DISH group. Additionally, Auðunsson et al. [23]. examined 5321 patients and reported that the prevalence of DISH increased with higher BMI. Conversely, the current study showed no correlation between the average patient BMI and the number of fused vertebral bodies present due to DISH, suggesting that ossification severity did not correlate with obesity in patients.

In terms of coexisting DM, the association with DISH is controversial at present. Some reports indicate that patients with DISH are significantly more likely to have coexisting DM [21,24,25], whereas others have found no association [22,26]. In the current study, HbA1c was used as a marker for DM. When the two groups matched for age and sex were compared, the HbA1c levels were significantly higher in the matched-DISH group than in the matched-non-DISH group. Since the HbA1c levels did not correlate with the number of fused vertebral bodies, the possibility of the coexistence of DM may be equally high for patients with and without severe ossification due to DISH. Therefore, when procedures such as surgery are performed, DM cannot be used as an indicator of the severity.

In addition, renal function tended to be slightly more impaired in the matched-DISH group; however, no significant difference was observed. Moreover, for the involvement of hypertension and metabolic syndrome, the current study showed no significant difference between the matched-DISH and matched-non-DISH groups. Likewise, no correlations were observed between these parameters and the number of fused vertebral bodies. These findings suggest that multiple comorbidities are not correlated to DISH or the fusion of vertebral bodies within the condition and, therefore, they cannot be used as an indication of disease presence or severity.

The limitations of the current study include its small sample size, a possible selection bias due to the inclusion of many patients with medical conditions, and the inability to analyze hyperuricemia, ischemic heart diseases, and cerebrovascular disorders, due to insufficient data.

## Conclusions

The study confirmed that DISH was more prevalent in the elderly and men. Additionally, we found that the condition was significantly more likely to coexist with obesity and DM. However, the extent of ossification progression present within the patients with DISH did not correlate with age, sex, BMI, or HbA1c levels. These findings mean that even DISH patients with less advanced ossification are likely to have both obesity and DM and should be given the same attention as those with advanced ossification.
